# Supplementary material for: Enhancing Classification Performance of fNIRS-BCI by Identifying Cortically Active Channels Using the z-Score Method
Source: Sensors (Basel). 2020 Dec 7;20(23):6995. doi: 10.3390/s20236995 (PMC7730208; doi:10.3390/s20236995)
Supplement: Supplementary file 1 [file sensors-20-06995-s001.pdf]

Table S1: Details of the selected channel using *t*-value and z-score method for MA task

	Mental Arithmetic (MA)				
	t-value method		z-score method		Total number of common channels
	Selected Channels	Total number of channels	Selected Channels	Total number of channels	
Sub1	1-11,13-36	35	10,12,17,25,27,33,35-36	8	7
Sub2	1,2,3,4,6,7,8,9,10,12,18,19,31, 33,34	15	4,6-8,10,13,15,19,22,24-25,30,33,34,36	15	8
Sub3	1-36	36	6,10,12,15,17,20-22,24-26,32-34	14	14
Sub4	1-35	34	1-2,4,10,-15,18,23-24,26-27,31-32,34	17	16
Sub5	1,5,7-8,23,28-30	8	1,4,9-12,16-18,21-24,26-27,32,35-36	18	2
Sub6	2-7,11,23,25-27	11	2-3,7,10-12,18,20-27,32	15	10
Sub7	1-2,8-12,14,18,20-21,26,30-34	17	1-9,12,15,17-18,20,23,26-27	17	8
Sub8	1-7,24,26-27,32	11	10-13,15,17,19,21-29,31-34	20	4
Sub9	1,6,19,24-28,31-33	11	1,3,5,7,11-15,17-18,21-23,25,28-29,31,34,36	20	4
Sub10	2-4,7,9,12,21,26,30	9	1,2,4,6,9-14,19-21,23-24,26-27,29,34	18	6
Sub11	2-3,7-8,10-36	31	5-13,18-21,26-27,29,33-35	18	17
Sub12	1-36	36	1,5-8,13,15,17,20-22,25-27,29,34	15	15
Sub13	13-14,16,34-36	6	2-8,10-14,21-26,29	18	3
Sub14	1-30,32-36	35	3,5,7,11-13,18,20-27,29,31-32,34	18	17
Sub15	5-8,10-15,17-20,22-23,25,27-31,33-36	26	3,7,10-12,15,18-27,29,3-34	20	15
Sub16	1-13,15-35	34	1-11,13,15,19-20,23,29,32	18	18
Sub17	1-3,5,7-36	34	3,10-12,15,17-27,29,32-34	20	20
Sub18	1-9,11-13,19,21,25-26,28,30-36	24	3,6,9-11,13,18,21,24,26-27,29-36	19	14

Sub19	11-12,14-15,18,21-23,29-30,33,35	12	2-10,13,21,23,26-27,29	15	5
Sub20	3,5-12,14-18,20-36	31	1-10,15,17,20,29,35,36	16	14
Sub21	10-12,19-22,24-27,31-32,34-35	15	1-4,9-12,17,20-21,26,29,31,33,35,36	17	9
Sub22	1-15,17,22,24-27,29,32-33,36	25	3-4,6,8,10-12,14,17,21-22,24-27,29,32-34	19	18
Sub23	2-10,12-13,15,17-23,25-28,31-36	29	4,10-12,15,17,19-22,24-27,29,31-33	18	16
Sub24	1-2,4-6,8-12,14,18,23,25,36	15	2,4,6,8,10-12,15,21,24-25,29-30,32-33,35-36	17	10
Sub25	1-12,15,17-36	33	4,14-15,17,19-27,29,32-33	16	15
Sub26	1,3,5,7,10-20,22-27,29-36	29	2-8,15,17-18,21-24,26-27,29,35-36	19	14
Sub27	1,2,4-9,16-17,19,29-30	13	1-2,4,10-12,15,18-21,24,26,29,31-33,35-36	19	5
Sub28	7,10,17,25-26,27,30	7	11-13,15-25,27,29-31,34-35	20	4
Sub29	1-5,7,9,17-18,20-22,24	13	1,6,8-14,19-20,22-23,27,29-32,34	19	4

Table S2: Details of the selected channel using *t*-value and z-score method for LMI task

	Left Motor Imagery (LMI)				
	t-value method		z-score method		Total number of common channels
	Selected Channels	Total number of channels	Selected Channels	Total number of channels	
Sub1	1-11,13-16,18-36	34	10-11,12,25,36	5	4
Sub2	6 -8, 10-12,15,18 -26,30,33-35	19	6,10,15,16-18,20,22-26,32-33	14	11
Sub3	4 6 7 8 9 12 22 27 31 33 34	11	3,7,15-17,19-22,24-26,28,32	14	2
Sub4	1, 6, 9, 13-14, 16, 24, 29, 36	9	1,4-7,10-14,16,18,22,23,26-27,35	17	5
Sub5	1-2, 4, 6, 9	5	4,5,9-12,16-18,21-28	17	2
Sub6	12,21	2	1,2,4,5,7-11,21-27	16	1
Sub7	1-9,24, 30	11	1,4,6-9,12-13,17-18,23-24,26-27,29,31	16	8

Sub8	2-8,11-12,14-15,17,20-24,27	18	4,6,10,12,15,17,19,20-23,26-27,29,31-33,34_35	19	10
Sub9	1-2,4-9,14,26,31,34,36	13	1-7,11,14-15,17-19,22-26,31	19	9
Sub10	2-4,6,8,12-15,19-20,22,23-25,27,32	17	1-2,4-14,19-,21,26,29,35	18	9
Sub11	1,3-9,16,18,23,36	12	7-11,14,17-22,24,26-27,31-32,35	18	4
Sub12	12,26,29,33	4	13-15,17,19-27,29,32-33,35	17	4
Sub13	14,17,22,25,27,29,33-36	10	1-12,14,19,21,24,26	17	2
Sub14	1,4,6,9,11,14-15,18,20-29,31-33, 36	22	3,9,11,13-14,19-27,31-32,34-35	18	14
Sub15	1-20,22-23,25-36	34	3,10-14,18-26,28,32-33	19	17
Sub16	1-9,14,16,18,23,30,36	15	4-10,12,14,19,21,23-24,26-,29-31,34	18	9
Sub17	1-9,11-12	11	6,11,14-15,19-27,30,32,34	16	2
Sub18	11,14,17,20-36	19	13-14,19-20,24-35	16	16
Sub19	12-13,17,19,23,25,27-30	10	1,3,6-9,13,15,18-19,21,23,26-27,29,32-33,35-36	19	5
Sub20	1,9,15-16,20,29-30	7	3,5-7,10,12-13,15-22,25,33,35	18	3
Sub21	1,3,7,17-18,22-29,31,34,36	16	4-7,10-12,17_24,26-27,32-33,35	20	8
Sub22	1-6,9,13,15,17,23-24,34	13	1,4,10-12,14-18,21-24,26,32-33,35	18	6
Sub23	1-18,20,22,24-26,28,30,34,36	27	4,10-12,15,17-18,21-22,24,26-27,32-33,35	15	15
Sub24	14,22,27-30,35-36	8	1-2,4-6,10-12,17,20-22,24,26,32-33,35	17	4
Sub25	1,13-16,18-36	24	1,5,11-17,19,21,22,24-27,33,35	18	15
Sub26	10,15,17-24,26,32-33	13	1,7,9,15,17-18,20,22-23,25,27-30,33,35-36	17	9
Sub27	1-2,4,6,10-11,23,25-26,28,36	11	10-13,15,18-22,24,26-27,31-35	18	3
Sub28	1-10,12-19,21-36	34	1,10,12-13,15,18-19,21-27,29,30,33	17	17
Sub29	12-16,23,25-26,34,36	10	1,2,4,6,8-15,18-19,22-23,27,32-33	19	6

Table S3: Details of the selected channel using *t*-value and z-score method for RMI task

	Right Motor Imagery (RMI)				
	t-value method		z-score method		Total number of common channels
	Selected Channels	Total number of channels	Selected Channels	Total number of channels	
Sub1	1-11,13-20,22-36	34	6,10-12,29-30,34-36	9	8
Sub2	1-9,12,14-18,20-22,25-27,29,30-31,33,35-36	27	4,6,9,14-15,17-19,21-27,29,32,36	18	14
Sub3	1,4,7,11-12,14,23,26-28,30	11	4,13-15,17,19-26,29,32-34	17	4
Sub4	3,5,10,12-13,21,24,26-27,29-31,34-35	14	1-2,4,10-16,18,22,24,26,31,36	16	6
Sub5	1-4,6,8-9,14,20,27,30	11	4-5,7,10-11,12,14-18,21-26,28	18	2
Sub6	1,3,6,11,15,27,36	7	1-2,4,8,9,11-13,18,20-27,35	18	3
Sub7	1-9,14,18,28,30,36	14	1-2,4,6-9,11,13,18-21,23,31,35	16	10
Sub8	1-36	36	10-12,15,17,20-23,25-27,29,31-34,35	18	18
Sub9	1,4,6,8-10,13-36	30	1-2,4-6,8-9,11-12,15-16,18-19,20-21,23-34,35	18	14
Sub10	1,10,12,16-18,20-22,26,31,34-36	14	1,4,6,10-16,20-21,23,26,29,34-35	17	10
Sub11	1-36.	36	1-8,10-12,20-21,24,31,35	16	16
Sub12	1,9,13-15,27,29-30,34-36	11	1,13-15,17,19-24,26,32-35	16	6
Sub13	10-11,13-14,16,23,30,34,36	9	1-12,19,20-21,24,35	17	2
Sub14	1,2,4,6,9-11,16,19	9	3,5,7,17-27,32,34-35	17	1
Sub15	2-3,5,7,11,13-15,17,28-30,36	13	10-12,17-21,23-27,31-33,35	17	2
Sub16	2-3,7-9,11,14-15,17-32,34-35	26	1-10,13,15-16,20-21,31-35	20	12
Sub17	2-8,10-12,17,20-31,33-35	26	10-11,13,15-16,18-27,31-34	19	14
Sub18	1,4-36	34	1,10-11,16,18,20-21,23,25-27,29-36	19	19
Sub19	1,3,5,7,9-20,23,25,28-31,33-36	26	3,5,7-8,10,12,15-17,22-23,25-26,28-30,33-35	19	17

Sub20	1-36.	36	3,5-7,13,15-21,25-27,29,32-33,35	19	19
Sub21	11,13-14,22-23,26,30-31,34-36	11	6-7,9,11-12,14,16-17,19-24,26,29,32,36	18	6
Sub22	10,12-14,16,19,26,30-32,34-35	12	6,9-12,14,16-18,21,23-24,26,29,31-34	18	10
Sub23	7-8,10,12,15-17,20-21,24-29,31-35	20	6,9-11,14,16-17,21-27,29,32-34	18	14
Sub24	22-23,25,27-29	6	1-8,10-12,17-18,20-22,24,26,32-33,35	21	1
Sub25	9-12,19,23,34-35	8	1-2,8,11-16,18-24,26-27,30,32-33	21	4
Sub26	10,15,17-18,20-24,26-27,31-33	14	1-2,8,15,17-18,20-27,30,32-33	17	12
Sub27	1-9,11-36	35	1,3-4,8,10-13,15,18-19,21,24,26-27,31-33,35	19	18
Sub28	1,10,19,22,26-28,31,34,36	10	8,10-12,16-18,21-25,27,29-33,35	19	5
Sub29	1-7,14,18,23,25	11	1,3,8-9,12-14,16-19,21,23-24,27,31-34	19	5
